# T cell receptor interactions with human leukocyte antigen govern indirect peptide selectivity for the cancer testis antigen MAGE-A4

**DOI:** 10.1074/jbc.RA120.014016

**Published:** 2020-06-12

**Authors:** Charlotte H. Coles, Catriona McMurran, Angharad Lloyd, Miriam Hock, Linda Hibbert, Marine C. C. Raman, Conor Hayes, Patrick Lupardus, David K. Cole, Stephen Harper

**Affiliations:** 1 Immunocore Ltd., Abingdon, United Kingdom; 2 Genentech, Inc., South San Francisco, California, USA; 3 Cardiff University School of Medicine, Cardiff, United Kingdom

**Keywords:** cancer-associated antigen, melanoma antigen-A4 (MAGE-A4), immuno-oncology, crystal structure, peptide-human leukocyte antigen (pHLA), surface plasmon resonance (SPR), T cell receptor (TCR), adaptive immunity, antigen recognition, cancer therapy, immunotherapy, antigen presentation, antigen recognition, cancer-associated antigen, MAGE-A4, peptide-human leukocyte antigen, T cell receptor

## Abstract

T cell-mediated immunity is governed primarily by T cell receptor (TCR) recognition of peptide-human leukocyte antigen (pHLA) complexes and is essential for immunosurveillance and disease control. This interaction is generally stabilized by interactions between the HLA surface and TCR germline-encoded complementarity-determining region (CDR) loops 1 and 2, whereas peptide selectivity is guided by direct interactions with the TCR CDR3 loops. Here, we solved the structure of a newly identified TCR in complex with a clinically relevant peptide derived from the cancer testis antigen melanoma antigen-A4 (MAGE-A4). The TCR bound pHLA in a position shifted toward the peptide's N terminus. This enabled the TCR to achieve peptide selectivity via an indirect mechanism, whereby the TCR sensed the first residue of the peptide through HLA residue Trp-167, which acted as a tunable gateway. Amino acid substitutions at peptide position 1 predicted to alter the HLA Trp-167 side-chain conformation abrogated TCR binding, indicating that this indirect binding mechanism is essential for peptide recognition. These findings extend our understanding of the molecular rules that underpin antigen recognition by TCRs and have important implications for the development of TCR-based therapies.

T cell–mediated immunity is essential during pathogen and cancer surveillance and plays a key role in autoimmunity and transplant rejection (1–5). T cells can interrogate the full cellular proteome via scanning of short peptides (a product of proteasomal degradation) presented on the cell surface by human leukocyte antigens (pHLA). T cell activation is tightly controlled by the clonally expressed T cell receptor (TCR) via direct recognition of its cognate pHLA. Thus, characterizing the molecular determinants that govern TCR-pHLA interactions is important for understanding the biology that underpins antigen discrimination by T cells, with obvious implications for the development of novel therapeutics. Accordingly, many studies have focused on characterizing the nature of functional TCR-pHLA interactions, particularly the biophysical (6–11) and structural parameters (4, 12–21) that govern T cell responsiveness.

Structural investigations have demonstrated that peptides are typically presented in an extended linear conformation, held in place by a comprehensive hydrogen-bonding network with the HLA binding groove. Aromatic residues within the HLA molecule, including Trp-167 and Tyr-84, restrict the size of the A and F pockets of HLA that accommodate the peptide N and C termini, respectively. Selectivity for different peptides is primarily driven by the N-terminal (peptide residue 1 or 2) and C-terminal peptide residue side chains, which are anchored by highly polymorphic HLA-binding pockets (22). Central peptide residues typically form a solvent-exposed motif for direct TCR contact (23).

TCRs normally bind to pHLA with a diagonal geometry over the center of the peptide, enabling the TCR complementarity-determining region (CDR) loops to engage solvent-exposed residues on both the peptide and the HLA helices (24). This orientation allows the somatically rearranged CDR3 loops to form the majority of direct peptide contacts, whereas the germline-encoded CDR1 and CDR2 loops generally form interactions with the HLA α1 and α2 helices (25). However, TCRs sometimes break these “rules of engagement” by binding toward the N or C terminus of the peptide (3, 26, 27), at extreme crossing or tilt angles (28, 29), or even in reversed polarity (30). These noncanonical binding modes can lead to further “breaking of the rules,” by enabling, for example, the germline-encoded CDR loops to make peptide contacts (3). Additionally, recent evidence has demonstrated that it is not only the TCR-accessible residues in the peptide that can determine TCR selectivity (31–33).

In this study, we solved the structure of a newly identified TCR in complex with an HLA-A*02:01 restricted melanoma antigen-A4 (MAGE-A4) peptide (GVYDGREHTV), hereafter referred to as A2-GVY. A2-GVY is a key oncology target because it is expressed by many tumors, including esophageal, head and neck, lung, ovarian, bladder, breast, lymphomas, and melanoma (34, 35). Our data demonstrated that the TCR bound in a position shifted toward the peptide N terminus. This enabled the TCR to achieve peptide selectivity for A2-GVY via a novel indirect mechanism whereby the TCR sensed the peptide features through an HLA residue, which acted as a tunable gateway or bridge. The altered side-chain conformation of this HLA residue was an antigenic feature for the TCR, correlating with the shifted binding footprint, to provide exquisite selectivity for the MAGE-A4 peptide N-terminal residue. Overall, our molecular characterization of TCR binding to a clinically relevant MAGE-A4 epitope extends our understanding of the general rules that underpin TCR antigen recognition.

## Results

### Identification of an HLA-A*02:01–restricted MAGE-A4 (GVYDGREHTV)–specific TCR and structural determination of its engagement mode

Following stimulation with peptide, we isolated an A2-GVY–specific TCR (GVY01) from CD8^+^ T cells from healthy HLA-A*02:01-positive donor peripheral blood mononuclear cells. The GVY01 TCR was identified from a panel of TCRs sequenced from A2-GVY tetramer-positive CD8^+^ T cells, and selected based on its ability to be expressed and refolded as a soluble molecule in bacteria and its ability to bind to A2-GVY in biophysical experiments.

**Figure 1.**
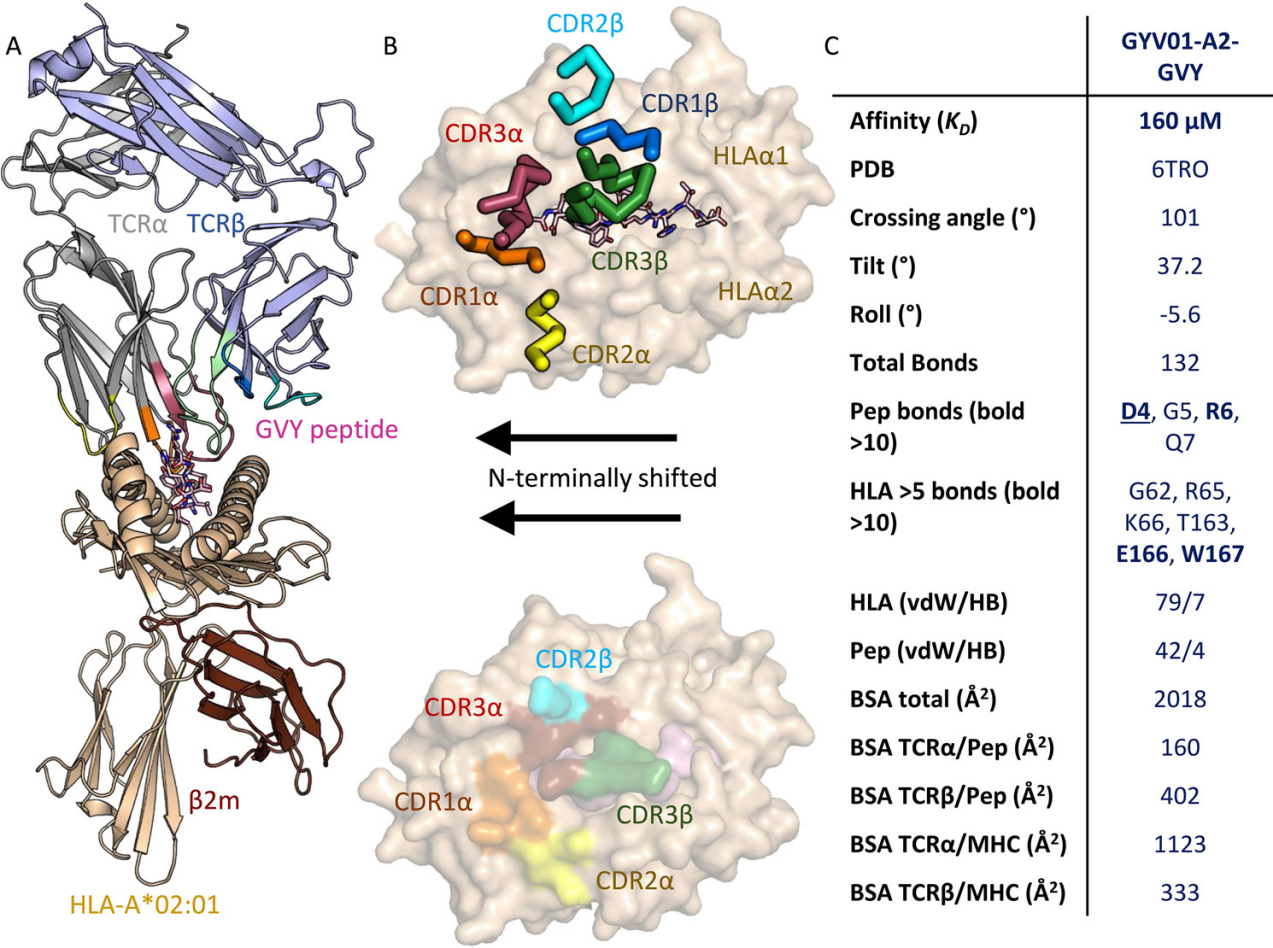
**Structural analysis of the GVY01-A2-GVY TCR-pHLA trimolecular complex.** *A*, structure of the GVY01 TCR (*gray* and *light purple cartoon*), in complex with A2 (*beige cartoon*)-GVY (*pink sticks*). *B*, *top*, the position of each TCR CDR loop (*ribbon colored* as *labeled*) is shown from above the HLA-binding groove (*beige surface*) with the peptide shown in *pink sticks*. *Bottom*, the HLA or peptide residues contacted by the GVY01 TCR are *colored* according to the TCR CDR loop forming the interaction (*colored* as *labeled*). The N-terminal shifted binding mode for the GVY01 TCR is indicated by the *arrows*. *C*, overview of TCR binding characteristics. Affinity was calculated using surface plasmon resonance. Crossing, tilt, and roll angles and buried surface area (*BSA*) were calculated using the Molecular Operating Environment program. Protein interactions were calculated by CONTACT from the CCP4 suite using a 4-Å cut-off for van der Waals (*vdW*) interactions and a 3.4-Å cut-off from hydrogen bonds (*HB*).

A structure of the GVY01-A2-GVY TCR-pHLA trimolecular complex was solved to 3 Å resolution (Table 1). The structure was characterized by well-defined observed electron density around the TCR-pHLA interface (Fig. S1). The GVY01 TCR bound to A2-GVY in a position shifted toward the peptide N terminus. Other TCR-pHLA interaction parameters were more typical (24): the TCR having a crossing angle of 101°, with the α-chain positioned over the HLAα2 helices and N terminus of the peptide, the TCR β-chain positioned over the HLAα1 helices and C terminus of the peptide, and the TCR CDR3α and CDR3β loops sitting directly above the peptide (Fig. 1, *A* and *B*).

**Table 1:** **Data collection and refinement statistics** Values in parentheses are for the highest-resolution shell.

	A2-AVY	GVY01-A2-GVY TCR-pHLA complex
**PDB accession code**	6TRN	6TRO
**Data collection**		
Space group	P2_1_	P6_4_ 2 2
Cell dimensions		
*a*, *b*, *c* (Å)	56.0, 80.7, 58.7	220.0, 220.0, 96.8
α, β, γ (degrees)	90.0, 115.1, 90.0	90.0, 90.0, 120.0
Resolution (Å)	48.31–1.35 (1.37–1.35)	67.91–3.00 (3.05–3.00)
*R*_merge_ (%)	4.7 (92.1)	9.4 (180)
*R*_pim_ (%)	2.8 (65.9)	3.7 (68.7)
*CC*_½_	0.999 (0.552)	0.998 (0.499)
*I*/σ*I*	12.2 (1.0)	13.5 (1.21)
Completeness (%)	99.5 (99.7)	98.6 (99.6)
Multiplicity	3.6 (2.9)	6.60 (6.80)
**Refinement**		
Resolution (Å)	48.31–1.35 (1.39–1.35)	67.91–3.00 (3.08–3.00)
No. of reflections	97,575 (7596)	26,360 (2023)
*R*_work_/*R*_free_	16.1/20.0 (29.2/32.5)	21.3/27.0 (34.1/37.3)
No. of atoms		
Protein	3190	6572
Water	439	23
Ethylene glycol	12	
*B*-Factors		
Protein	24.1	111.6
Water	36.8	73.8
Ethylene glycol	25.9	
Root mean square deviations		
Bond lengths (Å)	0.008	0.002
Bond angles (degrees)	1.467	1.203

Despite this N-terminal shift, the GVY01 TCR made conserved contacts (with respect to many other TCR-pHLA complex structures (24)) with two of the HLA restriction triad residues, Arg-65 and Ala-69, in addition to the central solvent-exposed residues in the peptide (Asp-4, Gly-5, Arg-6, and Gln-7) (Table S1 and Fig. 1*C*). However, its N-terminal shift abrogated its ability to make direct contacts with the third HLA restriction triad residue, Gln-155. Aside from these residues, the GVY01 TCR made most contacts with HLA residues Glu-166 and Trp-167, and peptide contacts were focused toward Asp-4, with Arg-6 making the second highest number of contacts with the GVY01 TCR (Table S1 and Fig. 1*C*). These interactions resulted in a total TCR-pHLA buried surface area of 2018 Å^2^ and ∼30% of the total contacts with the GVY peptide, which are both within the normal range for TCR-pHLA complexes (24).

**Figure 2.**
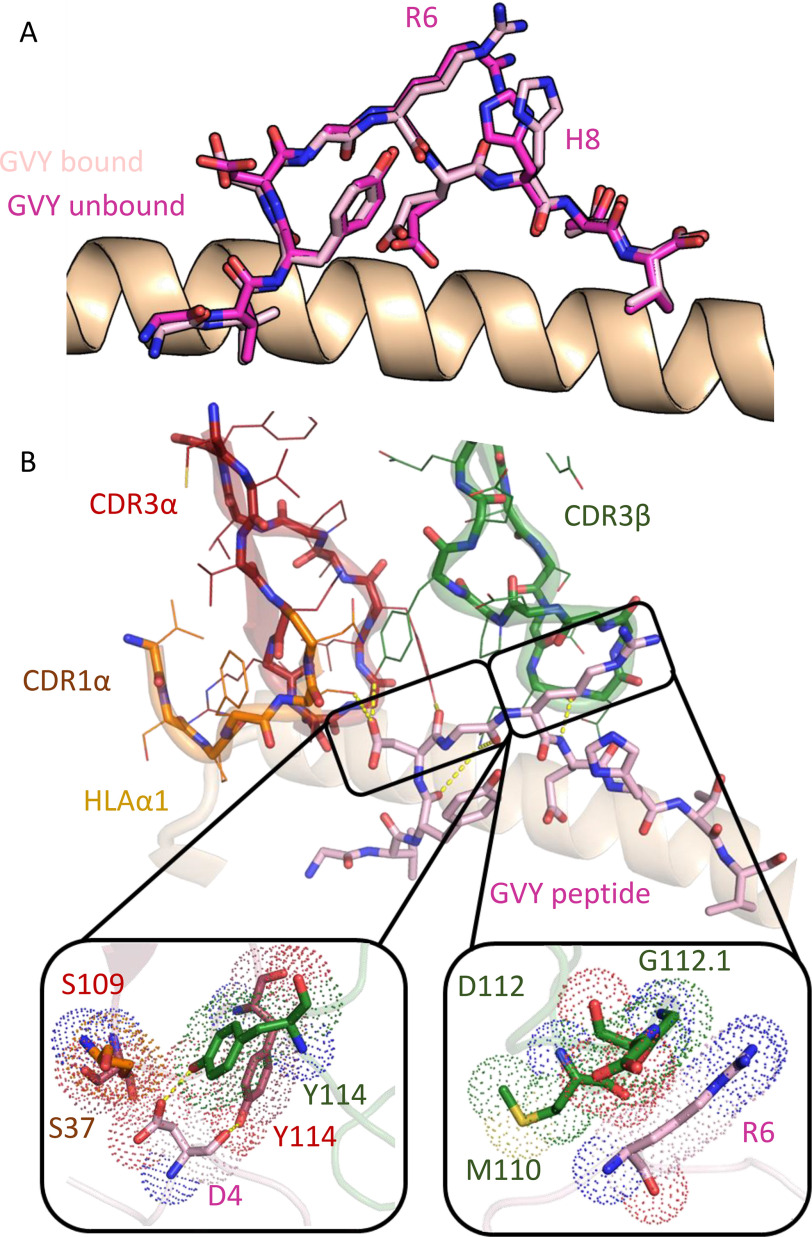
**The GVY01 TCR-peptide binding interface.** *A*, structural alignment of the TCR-bound (*pink sticks*) and unbound (*dark pink sticks*) A2-GVY co-complex (PDB accession code 1IF4). *B*, the GVY01 TCR CDR loops (*cartoon* and *sticks colored* as *labeled*) interacting with the A2-GVY peptide (*pink sticks*). *Panels below* show interactions between the GVY01 TCR CDR3 loops (*green* and *red sticks*) and peptide residues Asp-4 (*pink sticks*, *left-hand box*) or Arg-6 (*pink sticks*, *right-hand box*). *Yellow dashed lines*, putative hydrogen-bonding interactions. *Dotted spheres*, range of influence for van der Waals interactions.

Comparison of the previously published unligated A2-GVY co-complex (35) and the GVY01-A2-GVY TCR-pHLA trimolecular complex suggested some stabilization of the peptide residues Arg-6 and His-8 upon TCR ligation (Fig. 2*A*); however, significant conformational changes to A2-GVY were not apparent from the structural data. Importantly, the TCR-pHLA contact interface at Asp-4 and Arg-6 did not require major structural remodeling of A2-GVY (Fig. 2*B*), as has been observed for other TCR-pHLA interactions (31, 36, 37).

### The conformation of HLA residue Trp-167 is governed by position 1 in the peptide

**Figure 3.**
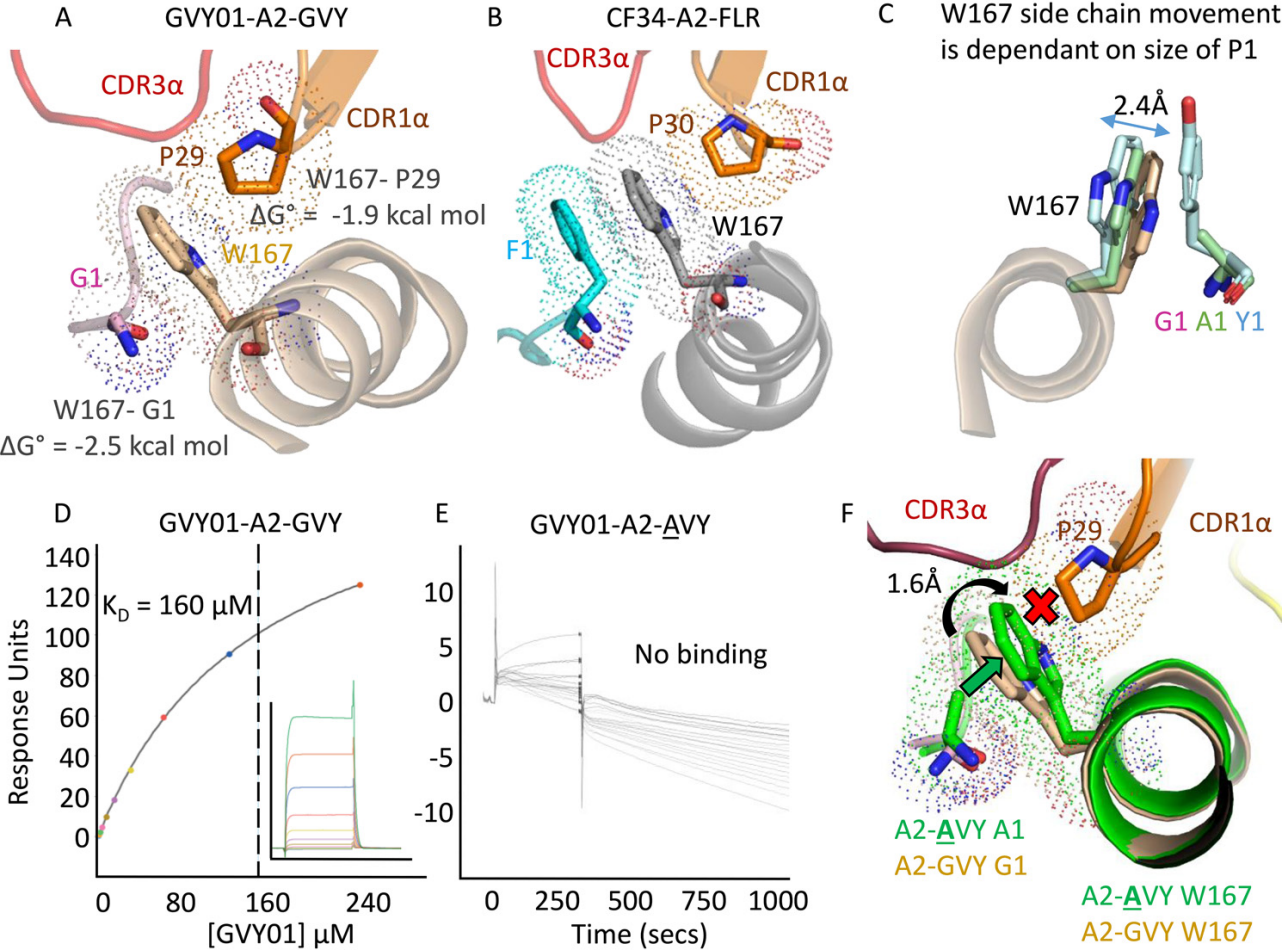
**GVY01 TCR-peptide selectivity guided via a molecular gateway mediated by HLA residue Trp-167.** *A*, indirect interaction between the GVY01 TCR CDR1α (*orange cartoon*) Pro-29 (*orange sticks*) and peptide residue Gly-1 (*pink sticks*) via HLA residue Trp-167 (*beige sticks*). The GVY01 TCR CDR3α is shown in a *red cartoon*. *Dotted spheres*, range of influence for van der Waals interactions. Binding energies were calculated using the Molecular Operating Environment program. *B*, indirect interaction between CF34 CDR1α (*orange cartoon*) Pro-30 (*orange sticks*), and peptide residue Phe-1 (*cyan sticks*) via HLA residue Trp-167 (*gray sticks*). *Dotted spheres*, range of influence for van der Waals interactions. CF34 CDR3α is shown in a *red cartoon*. *C*, the range of positions of Trp-167 evident in 10 different HLA-A*02:01 structures (*gray sticks*) with different peptide residues at position 1 (*multicolored sticks*). The *arrow* shows the range of movements of Trp-167 in the different structures. *D*, equilibrium binding of the interaction between the GVY01 TCR and A2-GVY. *Inset*, raw injection profiles for each concentration of the GVY01 TCR. *E*, equilibrium binding of the interaction between the GVY01 TCR and A2-**A**VY. *Inset*, raw injection profiles for each concentration of the GVY01 TCR. *F*, A2-**A**VY (*green cartoon* and *sticks*) was aligned with A2-GVY (*sand cartoon* and *sticks*) in the GVY01-A2-GVY TCR-pHLA trimolecular complex. The indirect interaction between the GVY01 TCR CDR1α (*orange cartoon*) Pro-29 (*orange sticks*) and peptide would be disrupted (represented by the *red cross*) in A2-**A**VY because of a 1.6-Å shift in Trp-167 (*green sticks* and indicated by the *green arrow*) toward the GVY01 TCR CDR1α Pro-29. *Dotted spheres*, range of influence for van der Waals interactions. The GVY01 TCR CDR3α is shown in a *red cartoon*.

Further inspection of the GVY01-A2-GVY TCR-pHLA trimolecular complex revealed that TCR CDR1α residue Pro-29 stacked closely against HLA residue Trp-167 (estimated Δ*G* = −1.9 kcal mol), which in turn packed against peptide position Gly-1 (estimated Δ*G* = −2.5 kcal mol), forming an indirect bridge between the TCR and peptide residue Gly-1 (Fig. 3*A*). This relatively strong indirect TCR-peptide interaction opened the possibility that the TCR might be able to sense the chemical properties of the peptide residue in this position. Reanalysis of the trimolecular complex structure of another N-terminally shifted TCR, CF34 (26), demonstrated a similar indirect interaction between the TCR CDR1α residue Pro-30 and peptide residue Phe-1 via HLA residue Trp-167, despite Trp-167 being shifted back toward the HLAα2 helices to accommodate the larger peptide P1 side chain in this structure (Fig. 3*B*). Thus, this observation is not isolated to the GVY01 TCR and may represent a more general TCR mechanism to sense peptide and achieve pHLA binding specificity.

We reanalyzed the structures of several other peptide-HLA-A*02:01 complexes with different amino acids at peptide residue 1 (Fig. 3*C* and Fig. S2). This analysis revealed that Trp-167 acts as a molecular gateway, altering its angle with respect to the HLAα2 helices, depending on the identity of peptide residue 1. Peptides with Gly-1 formed the first distinct family with respect to the position of Trp-167. For peptides with Ala at position 1, Trp-167 substantially altered its position to accommodate the slightly larger side chain, representing a second family. Ser, Thr, and Cys at peptide position 1 represented a third mechanism of distinct positioning of Trp-167, and three further families were apparent; the fourth with Val, Ile, Leu, and Pro; the fifth with Phe and Tyr; and the sixth with Glu, Asp, Arg, and Lys (Fig. S2).

These observations suggested that the GVY01 TCR might be sensitive to peptide alterations at position 1, despite making no direct contact with this peptide residue. The refolded soluble GVY01 TCR extracellular domain bound to A2-GVY with a *K_D_* of 160 μm as measured by surface plasmon resonance (Fig. 3*D*). This is within the binding affinity range of other tumor-specific TCRs (7, 8, 38). Binding affinity measurements of the GVY01 TCR to A2-GVY containing a G1A peptide mutation (the most conservative mutation possible) demonstrated a complete loss of binding (Fig. 3*E*). To explore this finding further, we solved the co-complex structure of A2-**A**VY at 1.35 Å (Table 1 and Fig. S1). A superposition of A2-GVY in the GVY01-A2-GVY TCR-pHLA trimolecular complex with the A2-**A**VY co-complex demonstrated that an Ala in position 1 would result in the CH2 of HLA residue Trp-167 shifting 1.6 Å toward TCR CDR1α loop residue Pro-29, disrupting the indirect interaction between the GVY01 TCR and the peptide through steric hindrance (Fig. 3*F*).

**Figure 4.**
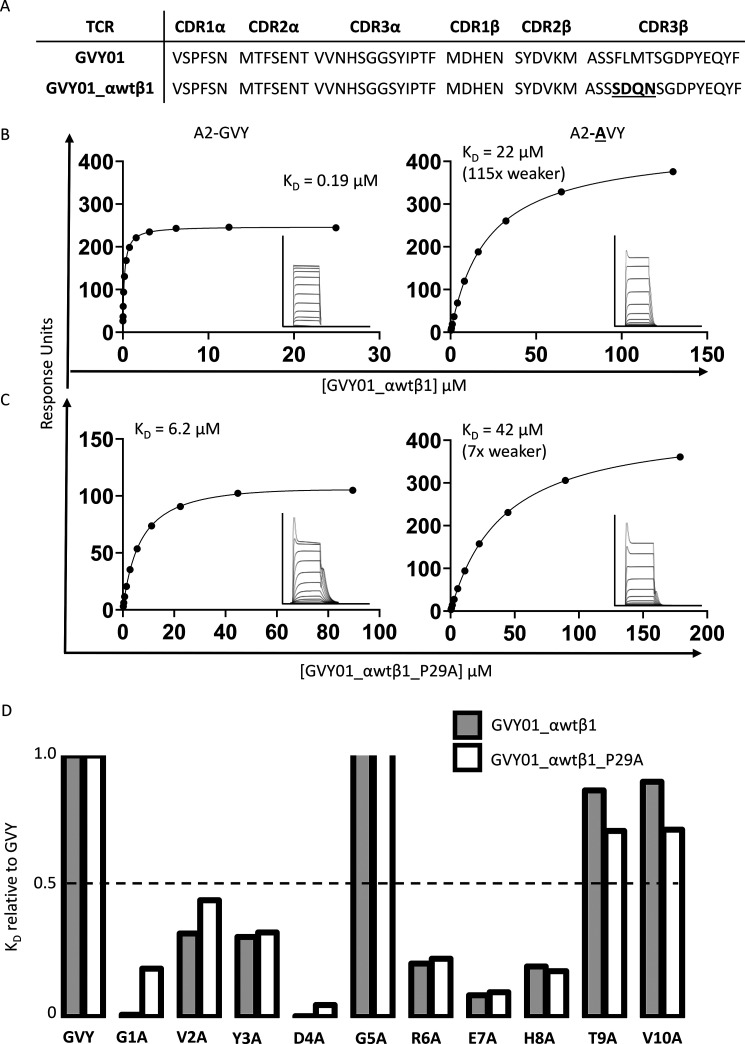
**Peptide selectivity of an affinity-enhanced GVY01 TCR via a molecular gateway mediated by HLA residue Trp-167 and the TCR CDR1α residue Pro-29.** *A*, sequence comparison of TCR CDR loops for the GVY01 TCR *versus* the GVY01_αwtβ1 TCR with mutated residues in the CDR3β loop *highlighted* in *boldface* and *underlined*. *B*, equilibrium binding analyses for the GVY01_αwtβ1 TCR interaction with A2-GVY and A2-**A**VY. *Insets* show the raw injection profiles for each concentration of TCR. *C*, equilibrium binding analyses for the GVY01_αwtβ1_P29A TCR interaction with A2-GVY and A2-**A**VY. *Insets* show the raw injection profiles for each concentration of TCR. *D*, summary of the binding affinity of the GVY01_αwtβ1 TCR or the GVY01_αwtβ1_P29A TCR to peptides containing individual Ala substitutions relative to WT GVY peptide.

A2-GVY is an important therapeutic target for a broad range of tumors. Thus, we used our previously described methods (39) to engineer an affinity-enhanced version of the GVY01 TCR, which contained four mutations in the CDR3β loop (Fig. 4, *A* and *B*). The selectivity of this affinity-enhanced GVY01 TCR (GVY01_αwtβ1) was determined by measuring its binding affinity to pHLA variants with individual peptide alanine substitutions. As with the GVY01 TCR, the GVY01_αwtβ1 TCR was also highly sensitive to changes at peptide position 1 (Fig. 4*B*). However, compared with the GVY01 TCR, the GVY01_αwtβ1 TCR bound to all of the peptide alanine mutants, including G1A, with a measurable affinity, thereby enabling a better comparison of the effects of point mutations in the TCR on the bridge between peptide residue 1 and Trp-167. For these analyses, we generated an alternative version of the GVY01_αwtβ1 TCR in which Pro-29 (the main TCR residue contacting HLA residue Trp-167) was mutated to Ala (GVY01_αwtβ1_P29A). For the GVY01_αwtβ1 TCR, a G1A peptide substitution reduced the binding affinity by 115-fold, whereas the GVY01_αwtβ1_P29A mutated TCR bound with only a 7-fold loss in affinity (Fig. 4*C*). Full analysis of Ala mutations along the peptide also confirmed these differences, in that, for the GVY01_αwtβ1 TCR, a G1A peptide substitution was the most detrimental to binding, whereas for the GVY01_αwtβ1_P29A TCR, G1A substitution made much less of an impact (Fig. 4*D* and Fig. S3). These data demonstrate that TCR residue Pro-29 was directly responsible for indirectly sensing the peptide via conformational changes in HLA residue Trp-167.

## Discussion

Cancer-reactive TCRs generally have weak affinity (7, 8, 38). This limitation, combined with the milieu of other factors that are known to hinder T cell responses to tumors, has led the field to develop new strategies to overcome these limitations and augment anti-tumor responses. One approach involves affinity-enhancing TCRs (39) for use in cellular therapies (40, 41) or as soluble bispecific T cell redirectors (42). However, understanding the binding selectivity of the TCR is fundamental to the development of effective and safe therapeutics (43–46).

Here, we solved a structure of the GVY01 TCR in complex with A2-GVY, a relevant target for MAGE-A4–positive tumors. Site-directed mutagenesis together with structural and biophysical analysis revealed a novel mechanism of TCR-peptide selectivity whereby the CDR1α loop of the TCR could sense the chemical properties of peptide residue 1 via a molecular gateway guided by a conformational change in HLA-A*02:01 residue Trp-167. Consistent with other studies comparing natural and affinity-enhanced TCRs (25, 37, 47–51), this binding selectivity was conserved for both the GVY01 TCR and an affinity-enhanced TCR variant, demonstrating that the fine selectivity in this system was maintained by the engineered version of the TCR.

Our findings add further evidence that conflicts with the classical view of TCR binding in which the somatically rearranged CDR3 loops contact the peptide, and the germline-encoded CDR1 and -2 loops contact the HLA. The mechanism presented here further highlights the importance of germline contacts in determining TCR-peptide selectivity but introduces a second factor through peptide-HLA contacts that shape epitope presentation to the TCR.

Importantly, there are a number of published examples where TCRs contact the peptide indirectly via HLA residue Trp-167, including in the trimolecular structure of the CF34 TCR (in complex with an HLA-A*02:01–restricted, Epstein Barr virus–derived peptide, FLRGRAYGL) (26) and in the trimolecular structure of the AS01 TCR (in complex with an HLA-A*02:01–restricted, Epstein Barr virus–derived peptide GLCTLVAML) (52), although the implications of this indirect peptide interaction for peptide selectivity were not considered in these studies. Additionally, it was found that the immunogenicity of an HIV reverse transcriptase peptide was increased 3-fold over WT by the substitution of Ile-1 for an aromatic residue (53). Immunogenicity was attributed in part to stabilizing π-π stacking interactions with Trp-167 in HLA-A*02:01, thereby increasing the binding *t*_½_. Although the mode of binding for this TCR was not described, these findings are consistent with our observation that Trp-167 plays a role in tuning TCR-peptide interactions. Furthermore, evidence for a role of Trp-167 in TCR recognition even exists in other species (54). Altogether, these observations imply a common indirect peptide sensing mechanism for TCRs through HLA residue Trp-167 binding that should be considered when studying the nature of TCR-mediated antigen recognition.

In addition to the implications for direct TCR-peptide selectivity, these data build on findings by ourselves, and others, showing that the peptide-HLA binding surface is more interdependent than previously thought. For example, we have shown that the C terminus of major histocompatibility complex α1 can open to accommodate additional residues, allowing the peptide to protrude out of the F-pocket (55), and that mutations in buried peptide residues can have knock-on effects that alter the conformation, and subsequent TCR recognition, of solvent-exposed peptide residues (32). Moreover, a number of studies have demonstrated that flexibility in both the HLA-bound peptide and the HLA helices can guide epitope presentation to T cells (56–60). Here, we add to these findings by showing that HLA residues can act as a molecular gateway, shifting side-chain conformations in relation to the characteristics of the peptide.

In summary, our data demonstrate that TCR-peptide selectivity can be achieved via indirect HLA contacts, guided by a peptide-dependent conformational change in the HLA molecule. Thus, we should consider the HLA surface as an extension of the peptide in instances where HLA conformation is the direct consequence of the peptide buried underneath. These findings have implications for our understanding of pHLA protein dynamics during TCR engagement and offer new insights into the mechanisms governing TCR-peptide selectivity, a major consideration for the rapidly emerging field of TCR-based therapeutics.

## Experimental procedures

### TCR generation, engineering, cloning, protein expression, and purification

Peripheral blood mononuclear cells, isolated from an HLA-A*02:01^+^ healthy donor, were stimulated with 1 nm GVYDGREHTV peptide. IFN-γ ELISpot assays were used to identify T cells that responded to MAGE-A4 peptide–pulsed T2 cells but not to T2 cells pulsed with an irrelevant peptide. T cells were restimulated, and single cell–sorted based on expression of the activation marker CD137. Sorted T cells were expanded for 2 weeks, followed by rescreening with an IFN-γ ELISpot assay. T cells that responded to MAGE-A4 peptide–pulsed T2 cells were isolated, and TCR chains were identified by rapid amplification of cDNA ends.

To obtain TCRs affinity-enhanced for A2-GVY, the WT GVY01 TCR was subjected to phage display as described previously (39). A panel of high-affinity TCRs were obtained with mutations in the α and/or the β chain (data not shown). The selected GVY01 clone contains mutations in the β CDR3 region improving the affinity from 160 to 0.17 μm.

The GVY01 TCR (or mutated variants), β2m, and HLA-A*02:01 heavy chains were cloned into the pGMT7 vector and expressed in the BL21 (DE3) Rosetta pLysS *Escherichia coli* strain as described previously (14). TCR constructs were designed to include the variable and constant domains of both chains (α and β) with an engineered interchain disulfide bond as described previously (3). The HLA-A*02:01 heavy chain was expressed with (for surface plasmon resonance experiments) or without (for crystallization screens) a biotinylation tag and refolded in the presence of β2m and the GVY peptide, as described previously (3). TCRs were refolded and purified using a previously described TCR refolding protocol (3).

### Biochemical assays

Surface plasmon resonance equilibrium binding analyses were performed using a BIAcore 8K^TM^ equipped with a CM5 sensor chip as reported previously (61). Biotin blocking was used as a negative control on flow cell 1, and analyses were all performed at 25 °C. For all experiments, ∼1000 response units of pHLA were coupled to the CM5 sensor chip surface. The TCR was then injected at concentrations ranging from 10× above and 10× below the known *K_D_* of the interaction at 10 μl/min. The *K_D_* values were calculated assuming 1:1 Langmuir binding (*AB* = *B* × *AB*_max_/(*K_D_* + *B*)), and the data were analyzed using a global fit algorithm (BIAevaluation^TM^ 3.1).

### Crystallization, data collection, data processing, and refinement

Crystallization trials, using 150 nl of protein solution plus 150 nl of reservoir solution in a sitting-drop vapor diffusion format, were set up in 2-well MRC crystallization plates using a Crystal Gryphon (Art Robbins) robot. Plates were maintained at 20 °C in a Rock Imager 1000 (Formulatrix) storage system. Diffraction quality crystals were grown for the GVY01-A2-GVY TCR-pHLA trimolecular complex in the following conditions: 1 m succinic acid, 0.1 m HEPES, pH 7.0, 1% (w/v) PEG MME 2K. Cross-seeding was used to obtain A2-GVY crystals. Briefly, 200 nl of protein solution plus 200 nl of reservoir solution drops were prepared across a 100 to 50% dilution screen of 0.2 m potassium sodium tartrate, 0.1 m bis-tris propane, pH 6.5, 20% PEG 3350. A 50-µl seed stock was prepared from HLA-A*02:01 crystals containing an unrelated peptide, using 100% reservoir solution in a Seed Bead^TM^ Eppendorf tube (Hampton Research). 100 nl of either undiluted or 1:3, 1:9, or 1:27 diluted seed stock was then dispensed across the screening experiment. Crystals were cryoprotected using a 30% solution of ethylene glycol and then flash-cooled at 100 K.

X-ray diffraction data were collected at Diamond Light Source (Oxfordshire, UK) at the I04-1 beamline at wavelength 0.9282 Å (A2-AVY) or the I03 beamline at wavelength 0.9763 Å (GVY01-A2-GVY TCR-pHLA complex). Diffraction images were indexed, integrated, scaled, and merged using XDS and XSCALE through the XIA2 data-processing suite. *CC*_½_ (62), *R*_pim_, and *I*/σ*I* statistics in the highest-resolution shell (criteria *CC*_½_ > 0.5, *R*_pim_ < 100%, and *I*/σ*I* > 1) were used to determine high-resolution cut-offs (Table 1). Molecular replacement was used to phase all crystal structures, using PDB entry 5E00 chains A and B (for HLA-A*02:01 and β2m), PDB entry 3HUJ chain E (for TCRα), and PDB entry 6AVF chain B (for TCRβ) as search models in Phaser (63). Scoring functions after placing the final nonpeptide containing molecule were as follows: GVY01-A2-GVY TCR-pHLA complex, TFZ 10.4, LLG 1872; A2-AVY, TFZ 17.2, LLG 934. Subsequently, 100 cycles of jelly-body refinement (σ 0.05, weighting term 0.001, and global noncrystallographic restraints) in Refmac (64) yielded *R*-factors as follows: GVY01-A2-GVY TCR-pHLA complex, *R*_work_ = 29.3, *R*_free_ = 31.7; A2-AVY, *R*_work_ = 39.1, *R*_free_ = 39.2. Manual model adjustment was performed in Coot (65), and the Refmac suite (64) was used for refinement. Stereochemical properties of all models were assessed using the PDB Validation Suite (66). Ramachandran statistics were as follows: GVY01-A2-GVY TCR-pHLA complex, 95% favored, 5% allowed, and no rotamer outliers; A2-AVY pHLA, 99% favored, 1% allowed, and no rotamer outliers. Full data collection and refinement statistics are given in Table 1. TCR residue numbering was assigned according to IMGT conventions (67); despite sequence gaps between chain D residues 29–36, 59–63, 68–74, and 110–112 and chain E residues 29–37, 58–63, 72–74, 81–83 plus a two-amino acid insertion between chain D residues 111 and 112, the TCRα and TCRβ chains are continuous in the variable domains. Crystallographic figures were created using PyMOL (68). All structural alignments were performed using Superpose (69). Buried surface area, estimated Δ*G* of interactions, and TCR docking geometry statistics were generated using MOE (Molecular Operating Environment, Chemical Computing Group). PDB accession codes were 6TRO (GVY01-A2-GVY TCR-pHLA complex) and 6TRN (A2-AVY).

## Data availability

Coordinates and structure factors were deposited in the Protein Data Bank with accession numbers 6TRN and 6TRO. The authors declare that all other data supporting the findings of this study are available within the article and the supporting information.

## Supplementary Material

Supporting Information
