# Supplementary material for: T cell receptor interactions with human leukocyte antigen govern indirect peptide selectivity for the cancer testis antigen MAGE-A4
Source: J Biol Chem. 2020 Jun 12;295(33):11486–94. doi: 10.1074/jbc.RA120.014016 (PMC7450119; doi:10.1074/jbc.RA120.014016)
Supplement: Supporting Information [file supp_295_33_11486__index.html]

T Cell Receptor interactions with Human Leukocyte Antigen govern indirect peptide selectivity for the cancer testis antigen MAGE-A4 — TCR-HLA interactions govern indirect peptide selectivity — T cell receptor interactions with human leukocyte antigen govern indirect peptide selectivity for the cancer testis antigen MAGE-A4 — TCR-HLA interactions govern indirect peptide selectivity — Supporting Information 

# T cell receptor interactions with human leukocyte antigen govern indirect peptide selectivity for the cancer testis antigen MAGE-A4

## Supporting Information

- Supplemental Figures - Supplemental Figures
