# Supplementary material for: T cell receptor interactions with human leukocyte antigen govern indirect peptide selectivity for the cancer testis antigen MAGE-A4
Source: J Biol Chem. 2020 Jun 12;295(33):11486–94. doi: 10.1074/jbc.RA120.014016 (PMC7450119; doi:10.1074/jbc.RA120.014016)
Supplement: Supporting Information [file supp_RA120.014016_160350_1_supp_545754_qbphq7.pdf]

CDR loop	Gene Usage	TCR residue	Peptide residue	MHC residue	Number of VdW (≤4 Å)	Number of H-bonds (≤3.4 Å)
CDR1α	TRAV10*01	Ser28$^{O\gamma}$		Glu166$^{O\varepsilon2}$	1	1
	TRAV10*01	Pro29		Glu166	7	
	TRAV10*01	Pro29		Trp167	10	
	TRAV10*01	Phe36^{O}		Thr163$^{O\gamma1}$	4	1
	TRAV10*01	Phe36^{N}		Glu166$^{O\varepsilon1}$	3	1
	TRAV10*01	Ser37		Thr163	1	
	TRAV10*01	Ser37$^{O\gamma}$	Asp4$^{O\delta2}$		2	1
CDR2α	TRAV10*01	Thr57		Ala158	1	
	TRAV10*01	Phe58		Glu161	2	
	TRAV10*01	Phe58		Gly162	4	
	TRAV10*01	Ser59		Ala158	1	
	TRAV10*01	Ser59		Glu161	2	
FWα	TRAV10*01	Asp83$^{O\delta1}$		Arg169^{NH1}	1	
	TRAV10*01	Lys85$^{N\zeta}$		Glu166$^{O\varepsilon2}$	2	1
CDR3α	TRAJ6*01	Ser109$^{N\zeta}$		Lys66^{O}	2	1
	TRAJ6*01	Ser109		Thr162	2	
	TRAJ6*01	Ser109		Trp167	1	
	TRAJ6*01	Ser109	Asp4		7	
	TRAJ6*01	Gly110		Gly62	4	
	TRAJ6*01	Gly110$^{N\zeta}$		Lys66^{O}	4	1
	TRAJ6*01	Gly110		Ala69	2	
	TRAJ6*01	Gly112		Gly62	1	
	TRAJ6*01	Gly112^{O}		Arg65^{NH1}	3	1
	TRAJ6*01	Ser113^{O}		Arg65^{NH1}	2	1
	TRAJ6*01	Tyr114		Lys66	1	
	TRAJ6*01	Tyr114^{OH}	Asp4^{O}		5	1
CDR2β	TRBV28*01	Tyr57		Arg65	17	
CDR3β	TRBD2*01/N	Met110	Gly5		1	
	TRBD2*01/N	Met110	Arg6		4	
	TRBD2*01/N	Met110^{O}	Glu7^{N}		6	1
	TRBD2*01	Thr111		Ala69	1	
	TRBD2*01	Thr111	Glu7		1	
	TRBD2*01	Gly112.1	Arg6		10	
	TRBD2*01/N	Asp112	Arg6		1	
	TRBJ2-7	Tyr114^{OH}	Asp4^{O}		5	1

Supplementary Table S1. **GVY01-A2-GVY contact table**

N = N-nucleotide insertion

*A 3.4Å cut-off was used for H-bonds and salt bridges and a 4Å cut-off was used for vdW.

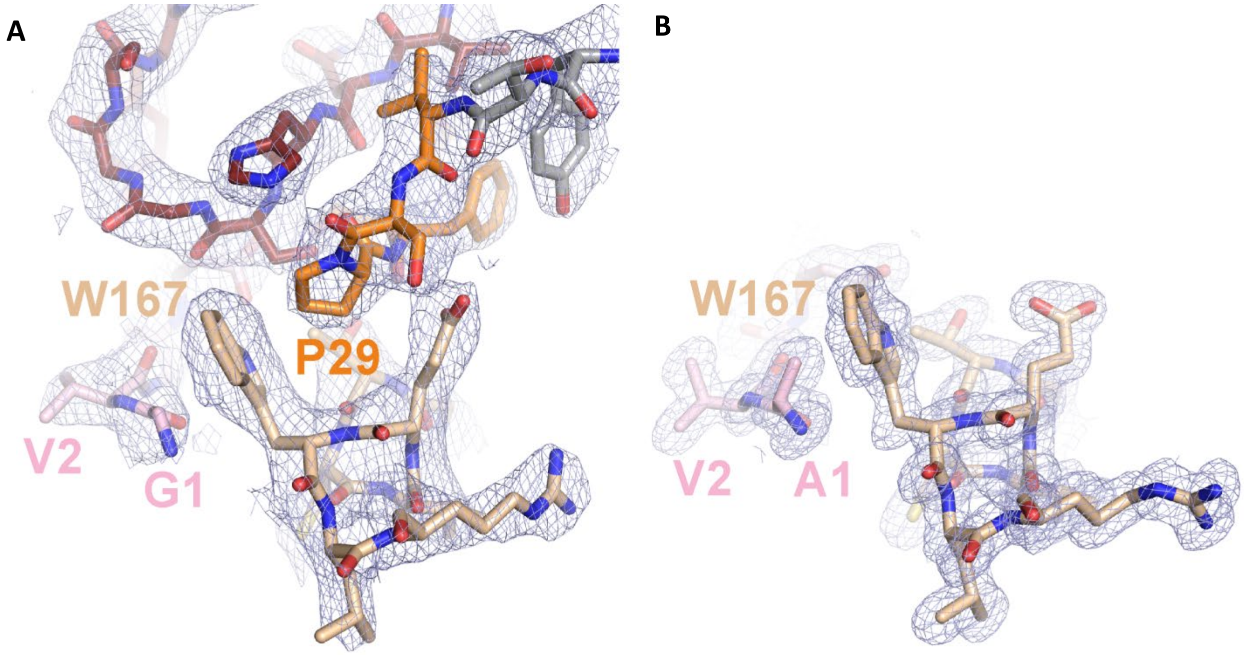

Supplementary Figure S1. Electron density quality illustrated for both **A)** GVY01-TCR-A2-GVY and **B)** A2-AVY crystal structures around the W167 gateway residue. 2Fo-Fc maps contoured at 1σ and carved within 2Å of the depicted residues are shown in blue. All protein residues are shown in stick representation: HLA heavy chain, wheat; peptide, pink; TCR CDR1α, orange; TCR CDR3α, maroon.

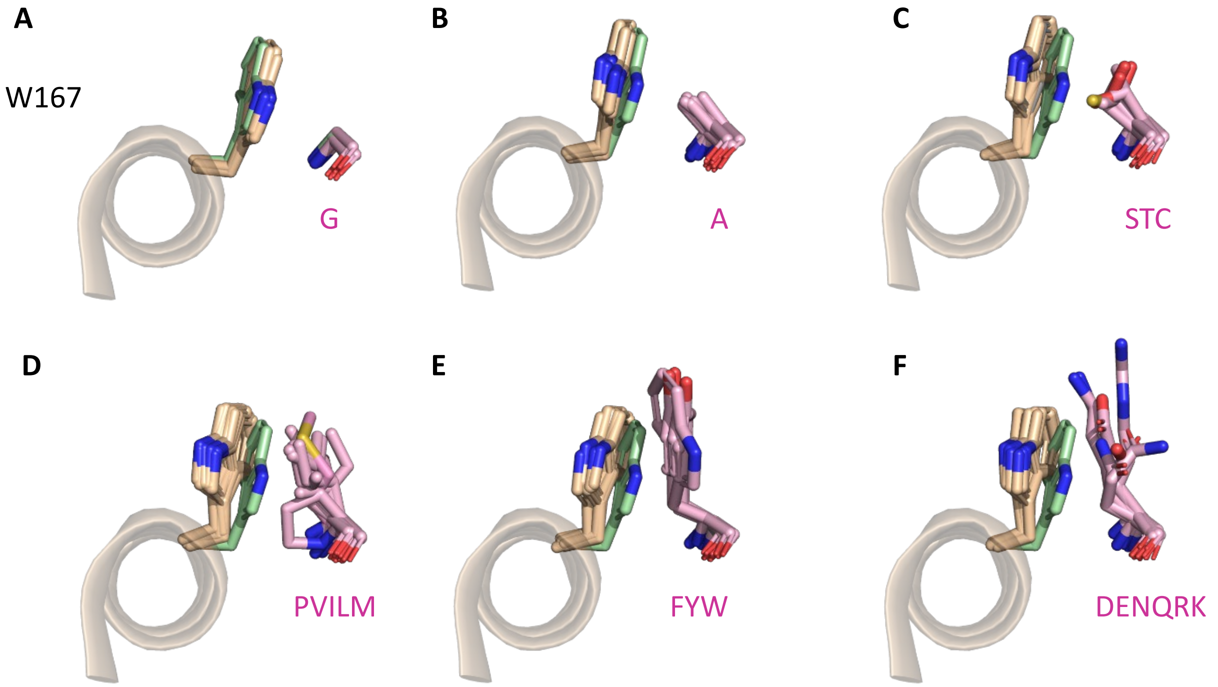

G

	phi Φ	psi Ψ	C- β	Chi χ1	Chi χ2
average Glycine	-61.6	-46.3	-121.2	-81.7	119.4
average Alanine	-72.6	-37.3	-125.4	-79.4	94.5
Small Polar (STC)	-70.6	-36.0	-125.1	-79.4	87.3
average small hydrophobics (ILV)	-75.4	-33.7	-127.5	-77.2	81.4
Aromatic (FY)	-72.5	-33.6	-125.7	-78.8	87.7
Large Hydrophillic (ENKR)	-76.2	-31.2	-130.1	-77.3	80.4

Supplementary Figure S2. The range of positions of Trp167 evident in different HLA-A*02:01 structures (wheat) are shown compared to Trp167 in MAGEA4 GVY 1i4F.pdb (greeen). Peptide residues at position 1 (pink) are Peptides with: **A)** Gly at position 1. **B)** Ala at position 1. **C)** Ser, Thr and Cys at position 1. **D)** Pro, Val, Ile, Leu, Met and at position 1. **E)** Phe, Tyr, Trp at position 1. **F)** Asp, Glu, Asn, Gln, Arg and Lys at position 1. **G)** Table detailing the different conformations of Trp167 depending on the identity of the amino acid at peptide position 1

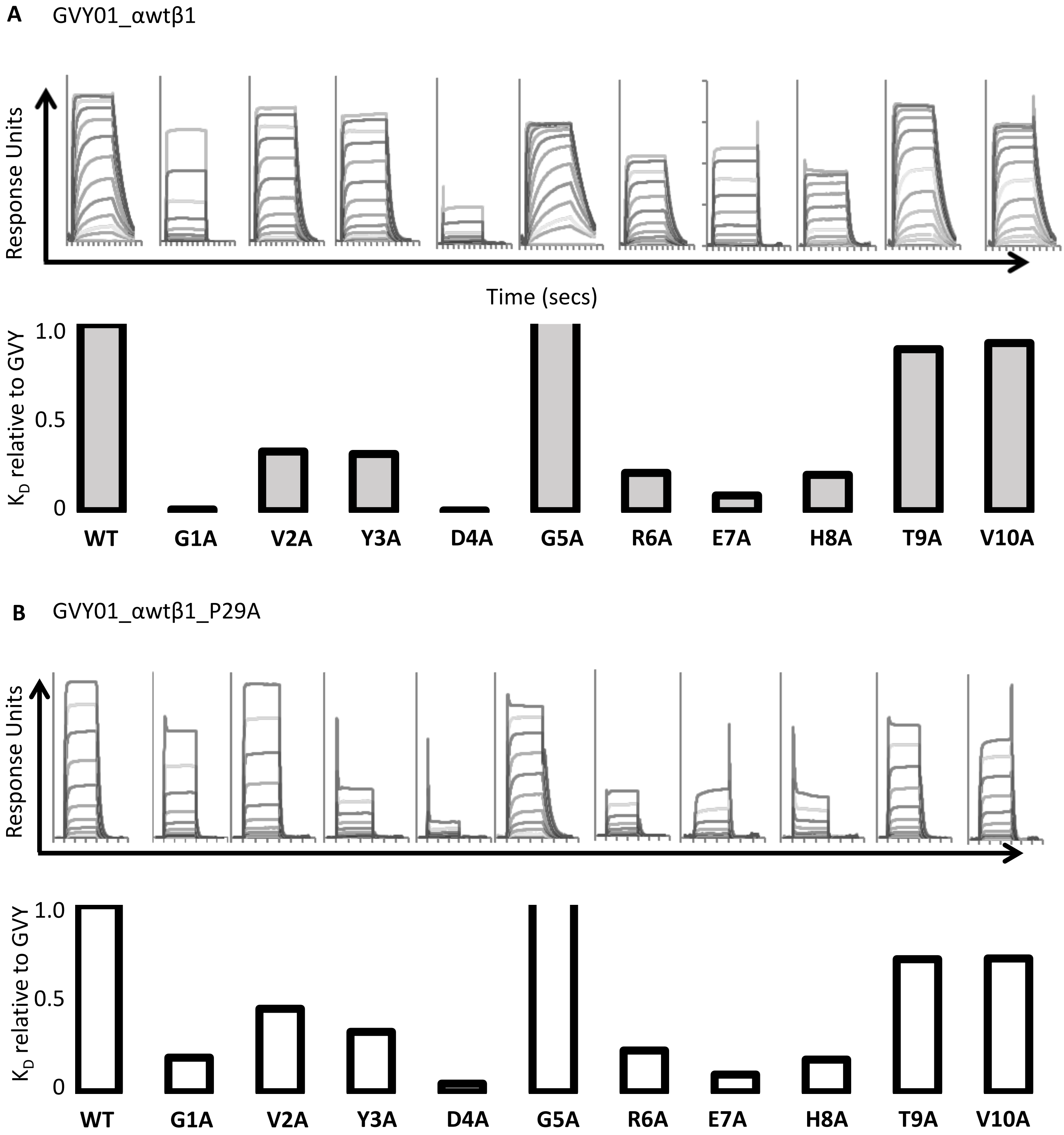

Supplementary Figure S3. A) Above: equilibrium binding raw injections, and below: relative difference in affinity of the interaction, between GVY01_αwtb1 and A2-GVY *versus* Ala substitutions across the peptide backbone **B)** Above: equilibrium binding raw injections, and below: relative difference in affinity of the interaction, between GVY01_αwtb1_P29A and A2-GVY *versus* Ala substitutions across the peptide backbone
